# BULL Database – Spanish Basin attributes for Unravelling Learning in Large-sample hydrology

**DOI:** 10.1038/s41597-024-03594-5

**Published:** 2024-07-06

**Authors:** Javier Senent-Aparicio, Gerardo Castellanos-Osorio, Francisco Segura-Méndez, Adrián López-Ballesteros, Patricia Jimeno-Sáez, Julio Pérez-Sánchez

**Affiliations:** 1 Department of Civil Engineering, Catholic University of San Antonio, Campus de los Jerónimos s/n, 30107 Murcia, Spain; 2 Department of Civil Engineering, Universidad de Las Palmas de Gran Canaria, Campus de Tafira, 35017 Las Palmas de Gran Canaria, Spain

**Keywords:** Hydrology, Environmental impact

## Abstract

We present a novel basin dataset for large-sample hydrological studies in Spain. BULL comprises data for 484 basins, combining hydrometeorological time series with several attributes related to geology, soil, topography, land cover, anthropogenic influence and hydroclimatology. Thus, we followed recommendations in the CARAVAN initiative for generating a truly open global hydrological dataset to collect these attributes. Several climatological data sources were used, and their data were validated by hydrological modelling. One of the main novelties of BULL compared to other national-scale datasets is the analysis of the hydrological alteration of the basins included in this dataset. This aspect is critical in countries such as Spain, which are characterised by rivers suffering from the highest levels of anthropisation. The BULL dataset is freely available at https://zenodo.org/records/10605646.

## Background & Summary

Large-sample hydrology (LSH) yields reliable insights into hydrological processes and models by leveraging comprehensive basin datasets. Recent review studies^1^ have underscored the fundamental role of these datasets in a wide range of hydrological investigations, including catchment classification^2^, assessments of terrestrial water storage and extreme events^3^, evaluations of hydrological models^4^, benchmarking^5^, parameter estimation^6^, regionalisation through machine learning algorithms^7^, analyses of human impacts on hydrology^8^, streamflow forecasting^9^, exploration of climate change impacts^10^, and assessments of data and model uncertainties^11^.

In recent years, several LSH datasets have been developed at a national scale. For instance, Addor *et al*.^12^ continued the work published by Newman *et al*.^13^ to create a dataset that included streamflow measurements, meteorological forcing data, and basin attributes for 671 watersheds in the contiguous United States.Other scientists extended this initiative in subsequentyears to develop similar databases in other countries, such as Chile^14^, the Great Britain^15^, Brazil^16,17^, Australia^18^, Central Europe^19^, China^20^, Iceland^21^, and Switzerland^22^. The vast number of hydrological databases published in recent years motivated the recently published work of Kratzert *et al*.^23^, which combined and standardised several LSH datasets into a global community dataset called CARAVAN. Recently, several datasets were published following the recommendations and codes provided by this initiative in Israel^24^, Germany^25^, Denmark^26^, and Spain^27^.

Despite being one of the driest countries in the European Union, Spain has the most irrigated croplands, accounting for 75% of total water resources consumption^28^. Paradoxically, the primary areas with irrigation are concentrated in the country’s most arid regions. This considerable imbalance between water resources and demands has prompted substantial investments in hydraulic infrastructure, leading to varying degrees of water resource exploitation across basins. Spain boasts the world’s largest reservoir capacity relative to its surface area; over 1,200 large dams (predominantly constructed in the mid-20th century) play a crucial role in the nation’s socio-economic development^29^. The extensive dam network has placed Spanish rivers among the most regulated globally, as evident from the GlObal georeferenced Database of Dams (GOODD)^30^ analysis. Notably, only 25% of the surface area of Peninsular Spain does not drain into one of the 823 largest dams recorded in the GOODD database, highlighting the pervasive influence of dams on Spanish rivers, which has resulted in the difficulty of finding flow gauging stations in a natural regime. Therefore, the need to characterise LSH datasets with the degree of hydrological anthropisation, as highlighted by Addor *et al*.^1^, is especially relevant in a country like Spain where a large number of dams makes Spanish rivers among the most regulated in the world, making it a great challenge to find those whose regime has not been altered.

However, recently published datasets following the recommendations of the CARAVAN initiative for Spain^27^ have not provided a detailed analysis of this issue. For instance, the degree of regulation is calculated in the CARAVAN initiative based on the Global Reservoir and Dam (GRanD) database developed by Lehner *et al*.^31^. Considering Spain, CAMELS-ES includes the degree of alteration and, the information provided by the Spanish Ministry of Environment on whether there are dams upstream of the gauging station. However, these criteria are not enough to identify hydrological anthropisation, since groundwater exploitation upstream of the monitoring station in some gauging stations is so important that the hydrological regime of the river is clearly altered despite the absence of dams. Hence, our work compared the observed flows of all study basins with the flows simulated by the national-scale hydrological model (Integrated System for Rainfall-Runoff Modelling, SIMPA)^32^ to identify basins with minimally altered hydrological regimes.

Precipitation is a pivotal factor in hydrological modelling since it significantly influences the accuracy^33^.This relationship exhibits nonlinearity despite its undeniable connection to various processes within the hydrological cycle, including the amount, intensity, and distribution. Nonetheless, precise precipitation assessment remains paramount for hydrological modelling, as it furnishes meteorological data crucial for hydrological studies^34^. Thus, ensuring reliable and accurate precipitation data at adequate spatial and temporal resolutions is imperative for scrutinising climate trends and effective water resource management^35^. Estimating precipitation across space poses challenges due to its spatio-temporal diversity and the complexity of involved physical processes^36^. However, recent advancements have seen notable strides in developing global reanalysis systems that combine observations of diverse variables with numerical weather predictions through data assimilation techniques^37^. ERA5-Land^38^ is one such reanalysis product the CARAVAN initiative recommends by the CARAVAN initiative for extracting meteorological forcing data. However, recent studies^39^ evaluating this product for Peninsular Spain have highlighted its poor detection capacity on the Mediterranean coast, especially during the summer. Other recent initiatives^27^ include the EMO-1 meteorological dataset^40^ developed for Europe at a higher resolution than ERA5-Land within the CAMELS-ES dataset. However, its performance has not yet been evaluated for Peninsular Spain. The BULL database includes the weather data for ERA5-Land and EMO-1 for all catchments, as well as the official grid of the Spanish State Meteorological Agency, whose performance has been shown as highly suitable for hydrological modelling^34^.

The main objective of this study was to present the BULL database, which was developed for application in large-scale hydrological studies following the CARAVAN initiative’s procedures. In addition, the 484 catchments were analysed to determine which had unaltered hydrological regimes. Moreover, data from three meteorological reanalysis products were analysed, considering precipitation and temperature estimation as well as their influence on the hydrological simulation using the Témez^41^ hydrological model. The BULL database expands on previous efforts by other authors, including hydrometeorological daily time series from 1951 to 2021. This database provides opportunities for identifying long-term trends for climate research over decades as well as conducting short-term local water cycle analyses in specific basins. The BULL database can also serve as a benchmark dataset for improved modelling and analysis tools in Peninsular Spain and holds potential for further extensions, such as refining the temporal resolution from daily to hourly, adding water quality and chemical data, and incorporating data from over 400 reservoirs available in the Official Gauging Station Network of Spain (ROEA).

## Methods

**Fig. 1:**
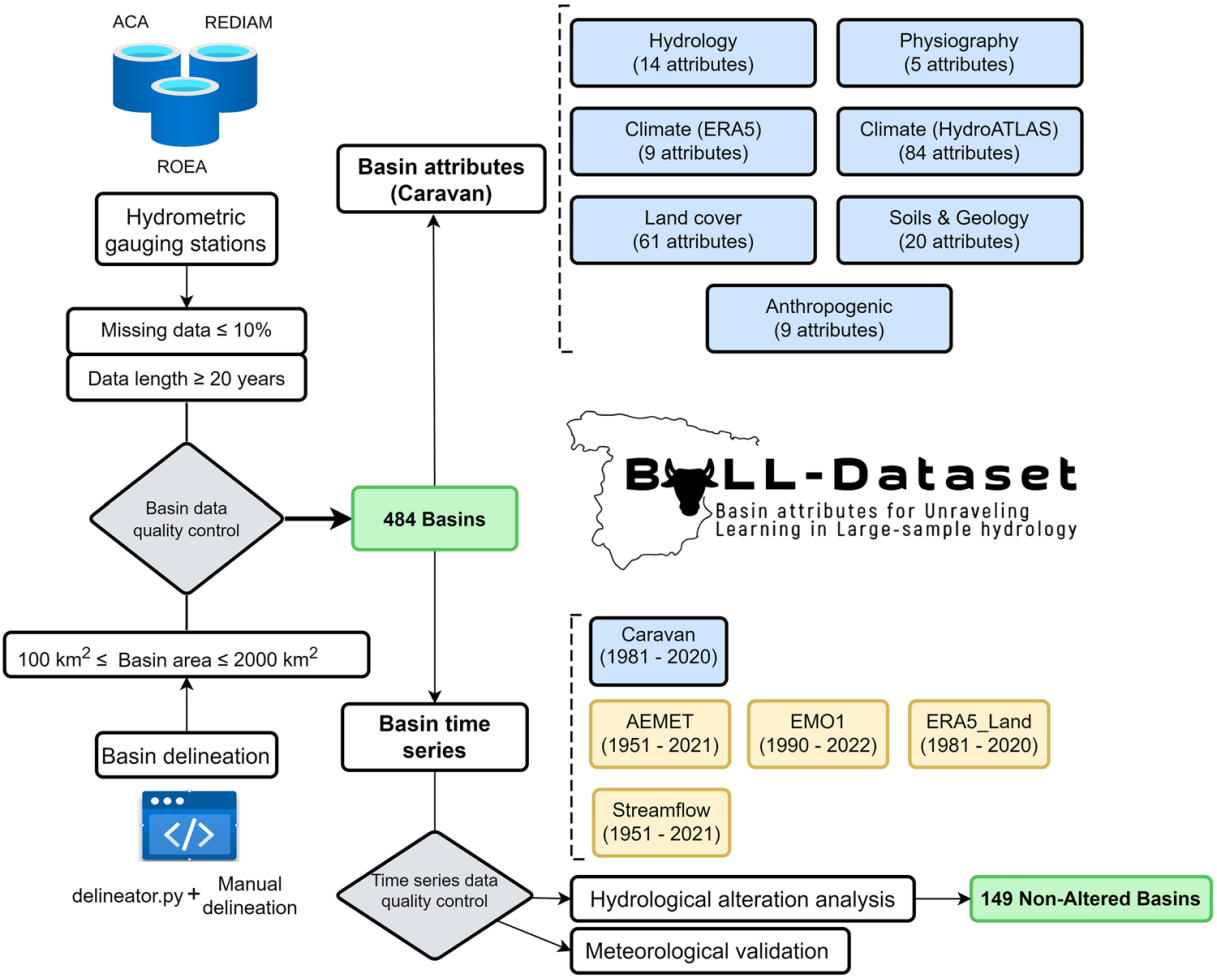
Schematic figure of the approach to generating the BULL dataset for Peninsular Spain.

The conceptual framework designed to build the BULL dataset is shown in a flowchart in Fig. 1. The first part of this study selected the basins to include in the BULL database. All available basins in the ROEA) were considered. Additional information from flow gauge stations provided by the Catalan Water Agency (ACA) and the Andalusian Environmental Information Network (REDIAM) was also obtained. These data were subjected to a series of selection criteria to obtain 484 basins. Secondly, the code provided by the CARAVAN initiative was used to extract meteorological forcing data from ERA5, and basin attributes to extend CARAVAN with data from 484 basins in Spain. Subsequently, an analysis was conducted to determine how many of these 484 basins were unaltered by comparing observational data with national-scale hydrological model SIMPA data. SIMPA is a distributed hydrological model that Spanish authorities use to evaluate water resources in natural regimes. It simulates the natural water balance and provides information about the main hydrological variables at a monthly time step and with a spatial resolution of 500 m. Finally, meteorological data from AEMET and EMO-1 products were extracted for all basins, analysing their performance, and simulating hydrological processes through the Témez hydrological modelto highlight the influence of the meteorological data in hydrological simulation.

### Basin selection and delineation

**Fig. 2:**
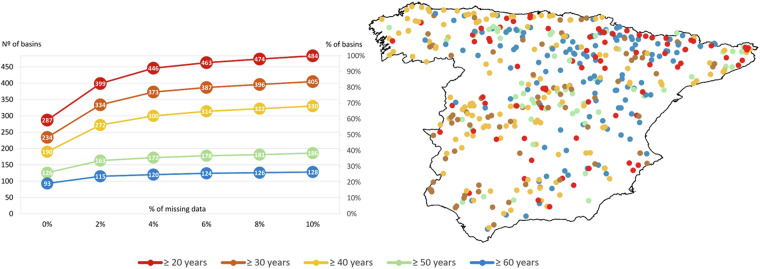
Analysis of missing data and time series length in streamflow stations: (**a**) the number and percentage of stations with different percentages of missing data across various time periods and (**b**) the length of the streamflow time series for each station.

Selecting basins for the BULL database began with data from the flow measurements available at the 1,634 stations registered in the ROEA, ACA, and REDIAM. Next, initial filtering excluded all flow measurement stations that only had data before 1951 since the available meteorological data from the Spanish Meteorological Agency began that year. As a result, the number of stations was reduced to 1,431. Then, the CARAVAN initiative’s recommended filter regarding the size of the basins was applied, discarding those smaller than 100 km² or larger than 2000 km², which reduced the number of available stations to 848. Subsequently, the percentage of gaps in the data from these stations was analysed, eliminating those with a percentage of gaps greater than 10%, reducing the number of available stations to 764. Finally, the minimum number of years with complete data available at these stations was studied, discarding all stations with less than 20 complete years of data, resulting in the total number of basins analysed in the BULL database, which was 484 (Fig. 2a). Two hundred twenty-nine of these basins have forest as the primary land use. Regarding size, 59% (n = 286) have an area between 100 and 500 km^2^, while only 6% of the basins (n = 28) have an area greater than 1500 km^2^. It is important to highlight that 287 basins, representing 60% of the total, have complete series without gaps for those 20 years. Additionally, approximately 19% of the basins have complete time series covering at least 60 years of observed daily runoff, while approximately 26% have 90% data coverage for that period. Figure 2b shows that the stations in the BULL dataset tend to have long-term gauging records, with the shortest record being 20 years, and more than half of the records (68% of the stations) being at least 40 years long. The distribution of stations according to the time-series length is uniform across the entire area, except for those with at least a span of 60 years, which are more abundant in the central-northern region.

Spain currently lacks a dedicated spatial database for extracting the geometrical attributes of gauged basins. Consequently, these basins’ dimensions were derived from previously acquired gauge data, designating them as the drainage focal points for Spanish gauged basins. The accuracy and spatial resolution of the digital elevation models (DEMs) were fundamental technical aspects during this phase^17^. Therefore, the choice was made to utilise the Delineator.py code^42^ to achieve this task, which employs hybrid methodologies, integrating vector- and raster-based approaches alongside data from MERIT-Hydro to delineate basins. These DEMs accurately depict terrain elevations at a 3-second resolution (approximately 90 metres at the equator), encompassing land areas between 90°N and 60°S for basin delineation purposes. Subsequently, geoprocessing techniques were employed to calculate the surface area of each delineated basin. The quality control process began by cross-referencing the information provided by the ROEA, ACA, and REDIAM, as well as the initial watershed delineation results obtained using delineator.py. Basins with significant differences in surface area were addressed by verifying the location of gauging points and adjusting the basin boundaries accordingly. Once the initial errors were rectified, the QGIS 3.23.3 command “fix geometries” was applied to ensure the integrity of the final basin geometries. Subsequently, the CARAVAN initiative scripts were applied to obtain each basin’s attributes.

### Methodological approach for identifying non-altered basins

As mentioned, Spain’s rivers exhibit a higher degree of anthropisation than other countries, mainly due to the large number of existing dams and the need to supply water to the entire Mediterranean Spain^43^. Therefore, the BULL initiative sought to analyse which basins included in the database had a natural or quasi-natural regime. Hence, the monthly observed flows of the 484 basins included in the BULL database were compared with the flows simulated by the national-scale hydrological model SIMPA, which Spain uses to calculate water resources in the various river basin plans developed under the framework of the European Water Directive^44^. To identify unaltered basins in the BULL database, we established a criterion of those whose observed flows compared to those of SIMPA produce a Nash-Sutcliffe coefficient^45^ (NSE) equal to or greater than 0.50. This NSE value is commonly used in hydrological modelling following Moriasi *et al*.^46^. A significant limitation of this approach was the assumption that the SIMPA model adequately reflected the natural regime flow series. The results of the model presented different sources of uncertainty due to input data error, model parameters, and model structure. However, as seen in the analysis of water resources for Peninsular Spain conducted by the Centre of Hydrographic Studies of the Centre for Studies and Experimentation of Public Works (CEDEX)^47^, the results were acceptable in most of the territory. Nevertheless, the efficiency decreased in the more arid areas of the Southeast with few unaltered gauging stations.

### Evaluation of reanalysis datasets for hydrological modelling

The meteorological data used to produce BULL were statistically validated by comparing and evaluating the robustness and accuracy of the datasets. AEMET was used as a benchmark^34,39,48^ to evaluate the performance of ERA5-Land and EMO1. The Spearman correlation coefficient (ρ), relative bias (RBIAS), root mean square error (RMSE), and Kling-Gupta efficiency (KGE) were calculated separately for each basin according to four meteorological variables: the total monthly precipitation and the monthly maximum, minimum, and mean temperatures. The equations of these statistics used for continuous analysis were described by Gomis-Cebolla *et al*.^39^. The coefficient ρ offers a robust assessment of the degree to which two variables exhibit a monotonic relationship, regardless of the data’s distributional properties. The RBIAS, expressed as a percentage, denotes the systematic bias in the estimates of precipitation and temperature, while the RMSE quantitatively represents the error characteristics between the different estimates of the variables and those taken as reference. KGE consolidates multiple statistical metrics into one measure, evaluating the model’s accuracy, variability reproduction, and temporal alignment with the reference data.

The Témez hydrological model was employed to validate the meteorological sources from a hydrological perspective. The Témez lumped rainfall-runoff model has been extensively employed in Spain for water resource management^34,49–51^ and in several other countries^52^. More details of the Témez model can be found in Pérez-Sánchez *et al*.^41^. The calibration process of the hydrological model was conducted to adjust its parameters to achieve an accurate streamflow simulation. AEMET data served as our reference dataset for calibration. The calibration involved a loss function based on the mean squared error (MSE), which compared observed and simulated streamflows. Once the parameters were calibrated, the streamflows were simulated using other available data sources. Optimisation was conducted using the least squares method with defined search bounds to identify the best model parameters. Specifically, the implementation in Python involved defining the loss function (MSE) and performing optimisation using the SciPy library’s minimise function with the L-BFGS-B algorithm^53^. To evaluate each hydrological model, the NSE, PBIAS, and RMSE observations’ standard deviation ratio (RSR) statistics were employed, following the criteria established by Moriasi *et al*.^54^, whose work provided comprehensive details on these widely used statistics for hydrological model evaluation. The RSR standardised the RMSE using observation standard deviation, providing an integrated error index.

## Data Records

The BULL dataset presented in this work^55^, encompassing 484 watersheds, can be accessed, and downloaded at https://zenodo.org/records/10605646. The dataset is organised according to the following folder structure:

- The attributes folder contains three CSV files obtained by using the CARAVAN script. One file, labelled “attributes_caravan_.csv”, comprises climate indices derived from ERA5-Land. The “attributes_hydroatlas_.csv” file encompasses attributes derived from HydroATLAS. In contrast, the “attributes_other_.csv” file incorporates other data relating to additional attributes such as the latitude and longitude coordinates, name, country of each gauge station and catchment area. The initial column in all attribute files is designated as “gauge_id”, featuring a unique basin identifier in the format “BULL_{id}”. Here, BULL corresponds to the source dataset, while {id} represents the basin ID defined in the original source dataset.
- A README.md file with the link to the scripts used is included in the code folder.
- The licenses folder encompasses licensing details for BULL and the incorporated dta. The README.md file in this directory provides general information about licenses and specific details about the source datasets used.
- The shapefiles folder contains a shapefile depicting the catchment boundaries of each basin included in the dataset. This shapefile was the basis for deriving the catchment attributes and ERA5-Land time series data. Each polygon in the shapefile is associated with a “gauge_id” field, containing the unique basin identifier.
- The timeseries folder comprises two subfolders, csv and netcdf, with the same structure and data—presented in CSV and netCDF formats. Within these subdirectories, five other subdirectories are labelled according to the source datasets, and individual files (CSV or netCDF) are allocated and encompass comprehensive time series data, including meteorological forcings, state variables, and streamflow. Moreover, the netCDF files incorporate metadata information such as physical units, time zones, and details regarding the data sources.
- The coordinate system used for shapefiles and netCDF files is the WGS 1984 Geographic Coordinate System (EPSG 4326).

## Technical Validation

### Validation of the identified unaltered basins

**Fig. 3:**
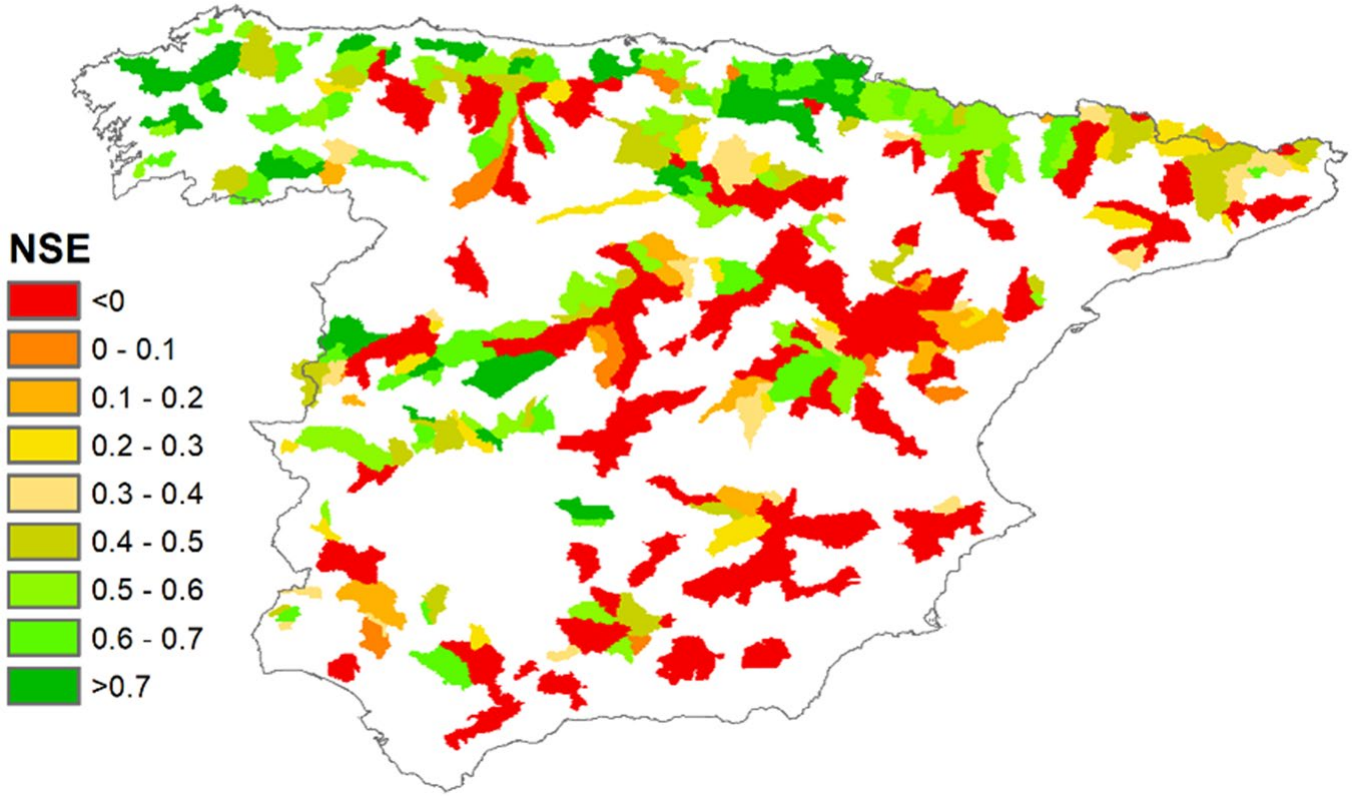
NSE values comparing observed data with SIMPA national scale hydrological model values.

Analysis of the distribution of the basins estimated to be in a natural regime revealed that most with a lower degree of anthropisation were located in Northern Spain, especially in Galicia, Asturias, Cantabria, the Basque Country, and the Western Pyrenees, as shown in Fig. 3. The basins with less altered hydrological regimes were abundant in areas influenced by oceanic climates. In contrast, those with more altered regimes were found in regions characterised by continental and Mediterranean climates. Radinger *et al*.^56^ detected variable flow patterns between and within geographical regions greatly influenced by climatic conditions. As expected, most of the basins analysed in the Mediterranean area exhibited high anthropisation. Leduc *et al*.^57^ repeteadly emphasised the anthropisation of water resources in the Mediterranean area. This analysis allowed validation of the information provided by ROEA that assesses whether basins are altered based on the presence of upstream reservoirs. The approach undertaken in this study confirmed that some of the basins the ROEA estimated to be unaltered were instead altered, because agricultural land use in these basins has significantly altered their hydrological regime. Thus, 149 unaltered basins were identified, which are of great utility for future large-scale hydrological studies in the Iberian Peninsula.

### Validation of reanalysis products using hydrological modelling

**Fig. 4:**
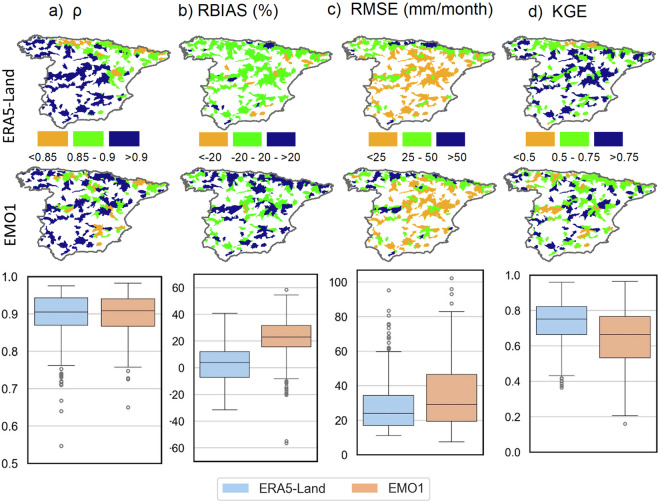
Spatial distribution and boxplot of ERA5-Land and EMO1 monthly continuous statistics: Spearman correlation (**a**), RBIAS (**b**), RMSE (**c**) and KGE (**d**) statistics.

Spearman correlation analysis, shown in Fig. 4a, illustrated a spatial gradient from the west-central regions (with the highest values) to the east (with the lowest values) for ERA5-Land. The highest RMSE values (Fig. 4c) were observed for ERA5-Land and EMO1 in the northern region. Gomis-Cebolla *et al*.^39^ found similar results indicating that the northern coast was one of the most critical regions in reanalysis modelling due to its performance. However, a more complez spatial pattern was observed for the rest of the statistics, which complicated straightforward spatial regionalisation. Regarding the correlation coefficient (Fig. 4a), the performance of ERA5-Land and EMO1 was similar, indicating that both were equally correlated with AEMET. However, ERA5-Land demonstrated better performance for RBIAS and RMSE compared to EMO1. In contrast, KGE showed a better performance for EMO1. Regarding monthly temperatures (i.e. the maximum, minimum, and mean), the correlation between all data sources was very high, with a median value higher than 0.98 for all temperatures. In addition, for RMSE performance, the median values were less than 1 °C.

Validation of these meteorological time series was conducted in a hydrological framework. Precipitation and temperature were fundamental inputs to hydrological models, where precipitation significantly influenced model accuracy^33^. In this study, the performance of three meteorological products was evaluated as input in a hydrological model for streamflow simulation. For this purpose, a common time period was selected during which climatological data from the three data sources (i.e. EMO-1, ERA5-Land, and AEMET) were available (1990–2020). This new time period considered which stations were identified as unaltered. The missing monthly streamflow data was less than 10%, identifying 87 stations in which the Témez hydrological model was applied.

**Fig. 5:**
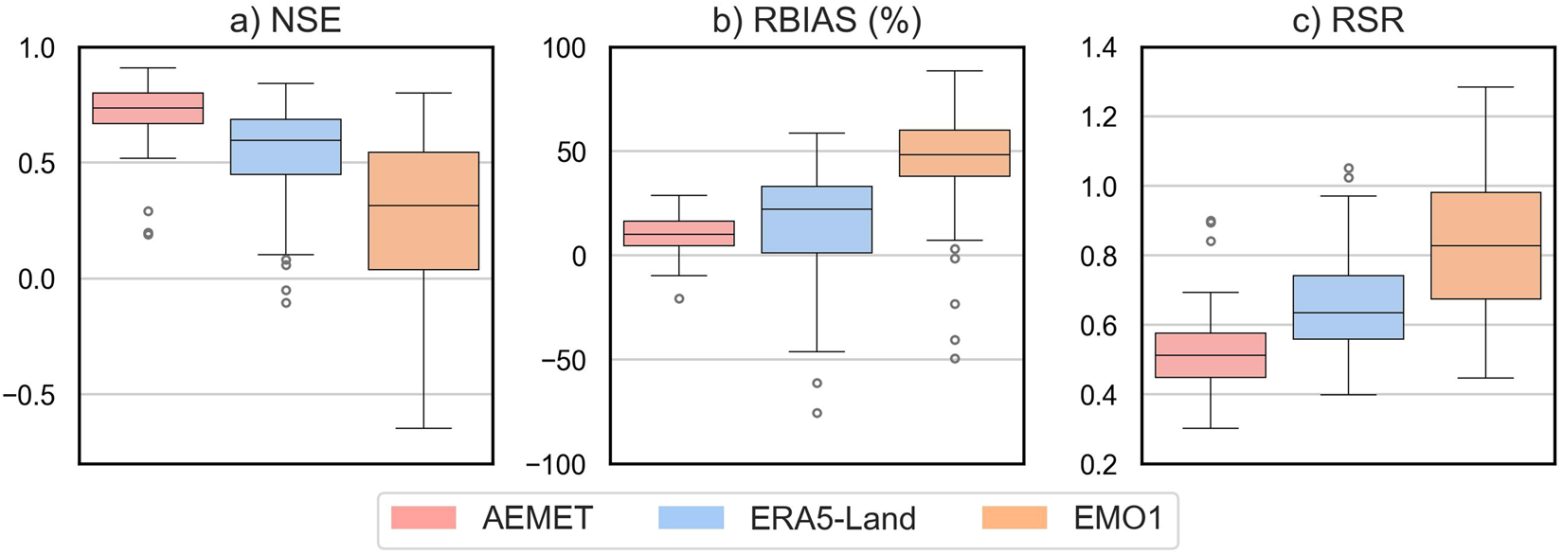
Boxplot of the monthly statistics from the streamflow simulation with the hydrological model using AEMET, ERA5-Land and EMO1 meteorological data: NSE (**a**), RBIAS (**b**) and RSR (**c**) statistics.

The model was calibrated using AEMET data, and the simulated streamflow was obtained using three precipitation and temperature products. The performance ofeach product (Fig. 5) was assessed by comparing it with the observed streamflows at the outlet of each basin. According to Moriasi *et al*.^54^, AEMET demonstrated performance exceeding satisfactory levels in nearly all basins. The worst models were observed with EMO1. The variability of the results obtained with ERA5-Land and EMO1 was much higher than with AEMET, with the highest dispersion observed with EMO1 data. The poorest performance was observed with EMO1, as depicted in Fig. 5, where the median values for NSE/RBIAS/RSR were 0.6/22/0.6 for ERA5 and 0.3/48/0.8 for EMO1.

## Data Availability

The code developed by Kratzert *et al*. in the CARAVAN initiative has been used for the calculation of the basin attributes (available at https://zenodo.org/records/6578598). The code used for the validation of the meteorological data including the coding of the Témez hydrological model are written in Python and are available at https://zenodo.org/records/10605646.
